# Is prophylactic lateral lymph node dissection needed for lower rectal cancer? A single-center retrospective study

**DOI:** 10.1186/s12893-021-01263-7

**Published:** 2021-05-26

**Authors:** Hiroka Kondo, Shigeki Yamaguchi, Yasumitsu Hirano, Masayasu Aikawa, Hiroshi Sato, Kojun Okamoto, Shinichi Sakuramoto, Isamu Koyama

**Affiliations:** Department of Gastroenterological Surgery, Saitama Medical University International Medical Center, 1397-1 Yamane, Hidaka-shi, Saitama, 350-1298 Japan

**Keywords:** Rectal cancer, Lateral lymph node dissection, Lateral lymph node metastasis

## Abstract

**Background:**

The effectiveness of prophylactic lateral lymph node dissection (LLND) in treating patients with lower rectal cancer remains controversial and has not been clearly established. Therefore, we aimed to retrospectively analyze the survival impact of prophylactic LLND in patients with lower rectal cancer.

**Methods:**

Data of 301 patients with lower rectal cancer (tumor’s lower edge on the anal side of the peritoneal reflexion) with clinical T3 disease and negative preoperative lateral lymph node metastasis, who underwent radical resection (R0) at our hospital between April 2007 and March 2017, were included in this study. Patients who received preoperative chemotherapy or radiotherapy were excluded. The relapse-free survival (RFS) and overall survival (OS) rates were compared between the dissection (prophylactic LLND, n = 37) and non-dissection (no prophylactic LLND, n = 264) groups.

**Results:**

Significantly fewer men and younger patients were noted in the dissection group than in the non-dissection group. Post-surgery 3- and 5-year RFS rates were 69.6% and 66.8% in the dissection group and 75.1% and 72.5% in the non-dissection group, respectively (5-year post-surgery RFS, p = 0.58). In the dissection and non-dissection groups, the 5-year OS rates were 86.5% and 79.7%, respectively (p = 0.29), and the 5-year cancer-specific survival rates were 88.9% and 86.0%, respectively (p = 0.29), with no significant differences. Lateral lymph node recurrence was observed in one (2.7%) and 10 patients (3.8%) in the dissection and non-dissection groups, respectively, and there was no significant difference between the groups.

**Conclusions:**

In this study, the effectiveness of prophylactic LLND was limited in patients with > T3 lower rectal cancer with no evidence of preoperative lymph node metastasis. Prophylactic LLND may not be necessary if there is no preoperative lymph node metastasis, even if the invasion depth is T3 or higher.

## Background

Rectal cancer is the tenth most deadly cancer, comprising 3.2% of all cancer-related deaths worldwide. The primary surgical modality for rectal cancers is proctectomy with lymph node dissection. According to the Japanese Colorectal Cancer Treatment Guidelines [1], lateral lymph node dissection (LLND) is recommended for rectal cancers in which the tumor’s lower edge is present on the anal side of the peritoneal inversion and the wall depth is deeper than clinical T3. Although, the survival benefit of LLND in preoperatively or intraoperatively diagnosed negative lateral lymph node metastasis is limited, it is still weakly recommended because of its role in suppressing local recurrence [1]. Nevertheless, the significance of bilateral lymph node dissection in lower rectal cancers will remain controversial, owing to the prolonged operation time and increased incidence of postoperative complications, unless it truly improves patient prognosis.

From the inauguration of our hospital in 2007, rectal cancer surgery was primarily an open procedure, and prophylactic LLND was performed for lower rectal cancers deeper than clinical T3. However, since then, rectal cancer surgery has gradually shifted towards the laparoscopic approach. Moreover, simultaneous advances in diagnostic imaging have improved the sensitivity for detecting lymph node metastasis preoperatively. Therefore, after June 2011, LLND was omitted in cases without evidence of lateral lymphadenopathy before surgery. Despite the recommendations and relevant developments, the effectiveness of LLND in treating patients with lower rectal cancer is still a subject of debate and has not been clearly established. Thus, we retrospectively analyzed and compared the survival outcomes of patients with lower rectal cancer who were treated with or without prophylactic LLND.

## Methods

This is a retrospective cohort study that focuses on the necessary of prophylactic LLND for rectum cancer. Data from a total of 301 patients with lower rectal cancer (lower tumor edge on the anal side of the peritoneal reflection) with clinical T3 disease, who were preoperatively diagnosed with negative lateral lymph node metastasis and underwent radical resection (R0) at our hospital from April 2007 to March 2017, were analyzed. Negative lateral lymph node metastasis was defined by the minor axis being shorter than 7.0 mm and a flat shape with even edges, as shown by computed tomography (CT) and magnetic resonance imaging. Patients who received preoperative chemotherapy or radiotherapy were excluded from the analysis. Also, if the surgeon accidentally found lymphadenopathy during the operation, such cases were excluded from the study. The patients were divided into two groups: patients who underwent prophylactic LLND (dissection group, n = 37) and those who did not undergo prophylactic LLND (non-dissection group, n = 264).

The post-surgery 3- and 5-year relapse-free survival (RFS) and overall survival (OS) rates were compared between the two groups. In addition, the local recurrence rate and its site, treatment after recurrence of lateral lymph node metastasis, and the incidence of complications, such as postoperative anastomotic leakage and self-catheterization, were retrospectively compared and examined. Cancer-specific survival was also compared between the two groups.

All statistical analyses were performed using the SPSS software package (SPSS version 25, IBM Corp., Tokyo, Japan). Chi-squared and Mann–Whitney *U* tests were performed to analyze the differences between the two groups. The cumulative cancer-specific survival rate was analyzed using the Kaplan–Meier method and log-rank tests. COX regression analysis was conducted to compare relapse-free survival (RFS) and overall survival (OS) rates. A p value < 0.05 was considered reflective of statistical significance.

## Results

Patient characteristics for the dissection and non-dissection groups are shown in Table 1. The male/female ratio and average age of the dissection (n = 37) and non-dissection groups (n = 264) were 21/16 and 197/67 and 60.38 ± 9.82 and 64.87 ± 11.52 years, respectively. Significantly fewer men and younger patients were noted in the dissection group, which also had a significantly lower American Society of Anesthesiologists physical status (ASA-PS) than in the non-dissection group. There was no difference between the two groups in terms of preoperative carcinoembryonic antigen levels and body mass index, the presence or absence of a history of laparotomy, and the proportion of preoperative T3 and T4 diagnoses. The proportion of patients who were preoperatively diagnosed with positive central lymph node metastasis was 70.3% in the dissection group and 51.1% in the non-dissection group (p = 0.03); however, there was no significant difference in the proportion of patients with pathological stage III disease between the groups (p = 0.37).

**Table 1:** Patient baseline characteristics

Characteristic	Dissection group (n = 37)	Non-dissection group (n = 264)	p value
Sex			
Male	21 (56.8)	197 (74.6)	
Female	16 (43.2)	67 (25.4)	0.02
Age (years)	60.38 ± 9.82	64.87 ± 11.52	0.01
	6.71 ± 5.45	21.18 ± 132.85	0.60
CEA (ng/mL)			
> 5.0	15 (40.5)	112 (42.4)	
≤ 5.0	22 (59.5)	152 (57.6)	0.83
	22.19 ± 3.44	23.42 ± 11.90	0.37
BMI (kg/m^2^)			
≥ 25	7 (18.9)	64 (24.2)	
< 25	30 (81.1)	200 (75.8)	0.48
History of laparotomy			
Yes	11 (29.7)	79 (29.9)	
No	26 (70.3)	185 (70.1)	0.98
ASA-PS			
1	27 (73.0)	101 (38.3)	
2	9 (24.3)	128 (48.5)	
3	1 (2.7)	35 (13.3)	< 0.001
Preoperative diagnosis			
Depth			
T3	32 (86.5)	219 (83.0)	
T4	5 (13.5)	45 (17.0)	0.59
Lymph node metastasis (central direction)			
Yes	26 (70.3)	135 (51.1)	
No	11 (29.7)	129 (48.9)	0.03
Surgery			
Approach			
Laparotomy	37 (100.0)	37 (14.0)	
Laparoscopy	0 (0.0)	227 (86.0)	< 0.001
Including anastomosis			
Yes	28 (75.7)	221 (83.7)	
No	9 (24.3)	43 (16.3)	0.23
Pathology			
Histopathology			
Well/moderate	36 (97.3)	236 (89.4)	
Other	1 (2.7)	28 (10.6)	0.10
Depth			
T1–2	6 (16.2)	79 (29.9)	
T3–4	31 (83.8)	185 (70.1)	0.08
Lymph node metastasis			
Central direction	20 (54.1)	122 (46.2)	0.37
Lateral	2	–	
Stage			
I/II	17 (45.9)	142 (53.8)	
III	20 (54.1)	122 (46.2)	0.37
Lymphatic invasion			
Yes	11 (29.7)	78 (29.8)	
No	26 (70.3)	184 (70.2)	0.996
Venous invasion			
Yes	27 (73.0)	193 (73.7)	
No	10 (27.0)	69 (26.3)	0.93
Nerve invasion			
Yes	19 (54.3)	105 (41.8)	
No	16 (45.7)	146 (58.2)	0.16
Postoperative adjuvant therapy			
Yes	15 (40.5)	92 (34.8)	
No	22 (59.5)	172 (65.2)	0.50

Data are presented as n (%) or mean ± standard deviation

*ASA-PS* American Society of Anesthesiologists physical status, *BMI* body mass index, *CEA* carcinoembryonic antigen

Bilateral prophylactic LLND was a laparotomy-based procedure in all patients in the dissection group, and a significant difference was observed between the method of approach in the dissection (laparotomy) and non-dissection groups (laparoscopy). There was no significant difference between the two groups in terms of the proportion of surgical procedures involving anastomosis (low anterior resection or intersphincter rectal resection), histopathological examination results (well/moderately differentiated adenocarcinoma or other; T1–2 or T3–4 disease; and the presence or absence of lymphatic invasion, venous invasion, and nerve invasion), and enforcement rate of postoperative adjuvant chemotherapy.

**Fig. 1:**
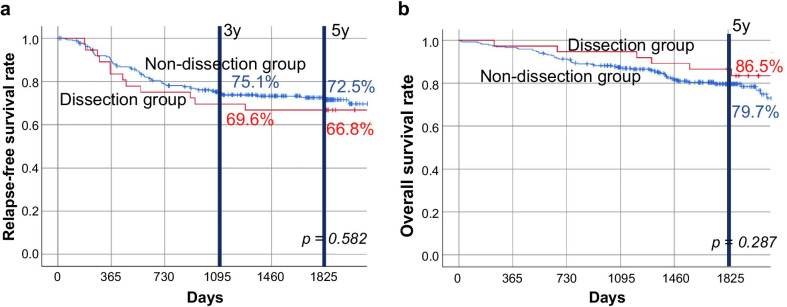
Relapse-free and overall survival. **a** Relapse-free survival. Kaplan–Meier curves of patients in the dissection group (red) and the non-dissection group (blue) indicating relapse-free survival rates. **b** Overall survival. Kaplan–Meier curves of patients in the dissection group (red) and the non-dissection group (blue) indicating overall survival rates

**Fig. 2:**
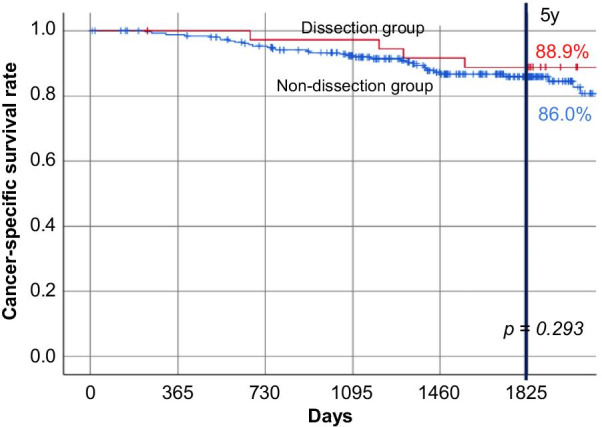
Cancer-specific survival. Kaplan–Meier curves of patients in the dissection group (red) and the non-dissection group (blue) indicating cancer-specific survival rates

There were two cases (5.4%) with positive lateral lymph node metastasis in the dissection group (Table 1). The RFS rates, 3 and 5 years after surgery, were 69.6% and 66.8%, respectively, in the dissection group, and 75.1% and 72.5%, respectively, in the non-dissection group (RFS 5 years post-surgery, p = 0.58) (Fig. 1a). The corresponding 5-year OS rates were 86.5% and 79.7% (p = 0.29), showing no significant difference (Fig. 1b). Furthermore, as for the background characteristics between the two groups, there were many young people in the dissection group and the ASA-PS tended to be good; hence, cancer-specific survival was also examined. The 5-year cancer-specific survival rate was 88.9% in the dissection group and 86.0% in the non-dissection group (p = 0.29), with no significant difference (Fig. 2). Cox regression analysis showed that male sex, high preoperative carcinoembryonic antigen levels, and pathological stage III disease had a significant effect on RFS. In addition to these factors, OS was affected by older age, open surgery, and non-adjuvant therapy. In either case (RFS or OS), the presence or absence of prophylactic LLND was not a significant factor (Table 2).

**Table 2:** Cox regression analysis of patient characteristics

Characteristic	Relapse-free survival			Overall survival		
	HR	95% CI	p value	HR	95% CI	p value
Male vs. female	0.561	0.323–0.973	0.04	0.474	0.243–0.927	0.03
Age (years)	0.992	0.971–1.013	0.44	1.036	1.008–1.065	0.01
CEA level (> 5.0 vs. ≤ 5.0 ng/mL)	0.485	0.309–0.762	0.002	0.420	0.249–0.709	0.001
Surgery (laparoscopy vs. open)	1.331	0.695–2.549	0.39	2.080	1.119–3.866	0.02
LLND (yes vs. no)	1.072	0.464–2.476	0.87	2.121	0.898–5.013	0.09
Anastomotic leakage (yes vs. no)	1.055	0.530–2.098	0.88	1.191	0.518–2.740	0.68
Histopathology (well/moderate *vs.* other)	0.846	0.385–1.861	0.68	0.470	0.185–1.191	0.11
Depth (pT3–4 vs. pT1–2)	0.681	0.380–1.220	0.20	0.557	0.276–1.124	0.10
pStage (3 vs. 1–2)	0.266	0.140–0.505	< 0.001	0.32	0.169–0.590	< 0.001
Adjuvant chemotherapy (yes *vs.* no)	1.219	0.668–2.227	0.52	2.23	1.167–4.272	0.02

*CEA* carcinoembryonic antigen, *CI* confidence interval, *HR* hazard ratio, *LLND* lateral lymph node dissection

**Fig. 3:**
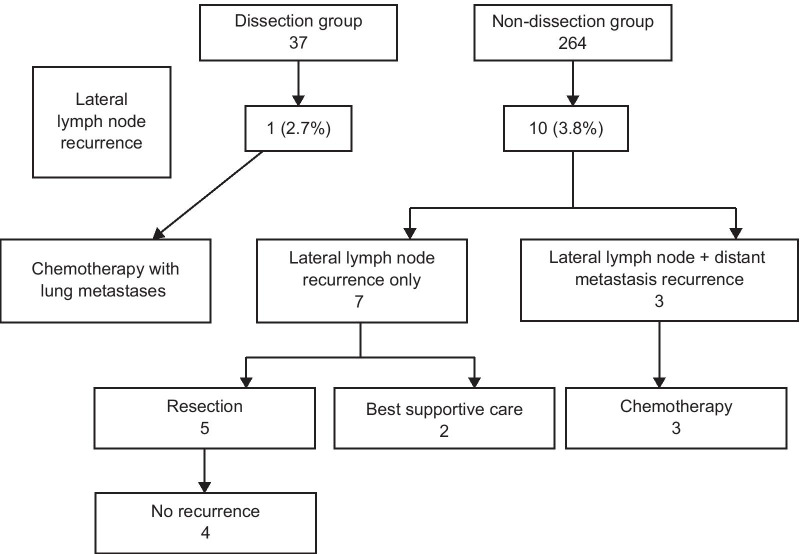
Treatment course after lateral lymph node metastasis recurrence

There was no significant difference in the overall recurrence rate (p = 0.48) or the rate of pelvic recurrence between the two groups (Table 3). Of the pelvic recurrences, lateral lymph node recurrence was observed in one case (2.7%) in the dissection group and in 10 cases (3.8%) in the non-dissection group; however, no significant difference was observed (p = 0.88). Of the seven patients in the non-dissection group who experienced initial recurrence as metastatic lateral lymph node involvement, lateral dissection was performed in five patients, and RFS was achieved in four patients. Moreover, three cases of distant lateral lymph node metastasis were noted, and none of them were treated by resection (Fig. 3). In the short-term, the mean operation time and blood loss were 317.92 ± 70.06 min and 467.68 ± 407.88 mL, respectively, in the dissection group and 262.03 ± 72.38 min and 79.79 ± 163.41 mL, respectively, in the non-dissection group. That is, the duration of surgery was significantly shorter, and the volume of bleeding was lower in the non-dissection group (Table 4). There was no significant difference in postoperative complications of Clavien–Dindo classification grade II or higher in six patients (16.2%) in the dissection group and 73 patients (27.7%) in the non-dissection group. Among postoperative complications, the incidence of anastomotic leakage was 14.3% in the dissection group and 14.0% in the non-dissection group. There was no significant difference in dysuria requiring self-catheterization between the dissection and non-dissection groups, respectively. The postoperative hospital stay was not significantly different between the two groups (Table 4).

**Table 3:** Recurrence rates of all patients

Variable	Dissection group (n = 37)	Non-dissection group (n = 264)	p value
All recurrence, n (%)			
Yes	12 (32.4)	71 (26.9)	
No	25 (67.6)	193 (73.1)	0.48
Pelvic recurrence, n (%)			
Yes	3 (8.1)	22 (8.3)	
No	34 (91.9)	241 (91.3)	
Unknown	0 (0.0)	1 (0.4)	0.93
Lateral lymph node recurrence, n (%)			
Yes	1 (2.7)	10 (3.8)	
No	36 (97.3)	254 (96.2)	0.88

Data are presented as n (%)” below the table

**Table 4:** Short-term postoperative outcomes

Variable	Dissection group (n = 37)	Non-dissection group (n = 264)	p value
Operation time (min)	317.92 ± 70.06	262.03 ± 72.38	< 0.001
Blood loss (mL)	467.68 ± 407.88	79.79 ± 163.41	< 0.001
Clavien–Dindo classification, n (%)			
Grade V	0 (0.0)	1 (0.4)	
Grade IV	0 (0.0)	0 (0.0)	
Grade III	4 (10.8)	37 (14.0)	
Grade II	2 (5.4)	35 (13.3)	
Grade I	8 (21.6)	28 (10.6)	
Grade 0	23 (62.2)	163 (61.7)	0.26
Anastomotic leakage, n (%)			
Yes	4 (14.3)	31 (14.0)	
No	24 (85.7)	190 (86.0)	0.58
Clean intermittent catheterization, n (%)			
Yes	0 (0.0)	8 (3.0)	
No	37 (100.0)	256 (97.0)	0.35
Postoperative hospital stay (days)	14.84 ± 9.42	14.82 ± 16.07	0.05

Data are presented as the mean ± standard deviation unless otherwise noted

## Discussion

In this study, we retrospectively evaluated the survival impact of prophylactic LLND in patients with lower rectal cancer. We found that the OS and RFS rates were not significantly different between the dissection and non-dissection groups. Therefore, the effectiveness of prophylactic LLND was found to be limited in patients with rectal cancer > T3 with no evidence of preoperative lymph node metastasis.

In Japan, a randomized controlled phase III trial (JCOG0212: ClinicalTrials.gov NCT00190541) [2] was conducted to evaluate the non-inferiority of mesorectal excision (ME) alone versus LLND plus ME in patients with clinical stage II/III rectal cancer without lateral lymphadenopathy. However, the study did not achieve its objective of demonstrating non-superiority. The endpoint, RFS, was similar between the two groups; however, patients who underwent ME alone had a significantly higher rate of local recurrence than did those who underwent LLND plus ME [2]. A subsequent study [3] has shown that LLND is more effective in clinical stage III patients than in clinical stage II patients. Currently, according to the Japanese Colorectal Cancer Treatment Guidelines, LLND is strongly recommended for lower rectal cancer deeper than T3 with lymphadenopathy, and weakly recommended for cases without [1]. In contrast, a study [4] has reported that prophylactic LLND does not contribute to the recurrence or survival rates, which is consistent with the results of our study. In other words, the postoperative survival and recurrence rates of patients with rectal cancer who have a preoperative diagnosis of negative lateral lymph node metastasis were comparable between those receiving and those not receiving prophylactic LLND. In our study, although the RFS rate was slightly lower in the dissection group than in the non-dissection group, the OS rate was slightly higher in the former. Since the average age of the dissection group was significantly younger than that of the non-dissection group, treatment after recurrence may have been adequate in many cases. In addition, when cancer-specific survival was examined to exclude the effects of other diseases in the elderly group in which dissection was omitted, the difference between the two groups almost disappeared. There was no difference in the local recurrence rate between the two groups. There was no significant difference in the lateral lymph node recurrence rate; however, the rate was slightly higher in the group in which dissection was omitted. These results suggest that LLND does not eliminate lateral lymph node recurrence, and that lateral lymph node recurrence in the non-dissection group is often curable.

There are several reports showing that LLND affects urination and sexual dysfunction [5]. This study compared voiding dysfunction with and without self-catheterization. The incidence of voiding dysfunction was very low in both groups, and no cases required self-catheterization after LLND. It has been reported that the degree of urination and sexual dysfunction can be reduced by preserving the autonomic nerve [6–9], and that there is no difference in the frequency of urination and sexual dysfunction with or without LLND [10, 11]. The risk of dysuria may be considerably low with current nerve preservation and LLND techniques, but the occurrence of dysuria in the JCOG0212 trial was not related to the presence or absence of LLND, and the volume of bleeding was a risk factor [12]. As for male sexual dysfunction, LLND did not have an effect, and age was identified as a risk factor [11]. We can infer that LLND does not invariably cause complications, but it may be associated with an increased risk of bleeding.

Additionally, several risk factors for positive lateral lymph nodes have been reported previously. Preoperative risk factors include age, sex, tumor location, depth of invasion, central nodal involvement, and lateral node size [13–23]. Studies on the size of lymph nodes in terms of the major or minor axis and various lengths have been conducted; however, in this study, lateral lymph nodes with minor axes shorter than 7.0 mm, as identified using CT and magnetic resonance imaging, constituted the criterion for negative metastasis, and many institutions in Japan now use this criterion. Despite implementing this criterion in two cases, which accounted for 5.4% of the dissection group, lymph node metastasis was histologically confirmed in the lateral lymph node, which points to the need for further evaluation of the optimum imaging modality and lymph node size and shape criteria for identifying lateral lymph node metastasis. Tumor marker monitoring every 3 months and CT examination every 6 months were performed in this study. In the 10 patients with lateral lymph node metastasis who did not undergo prophylactic LLND, the mean time to recurrence was 635.8 (102–1661) days. Since the time to recurrence was quite long, these results emphasize the importance of not neglecting surveillance until 5 years after surgery.

Finally, in this study, anastomotic leakage did not affect RFS and OS. Some previous reports, like our study results, indicate that there is no association between anastomotic leakage and local recurrence [24, 25], while others point to an increase in the local recurrence rate in patients with anastomotic leakage [26, 27]. As for the risk factors for anastomotic leakage, "being a man" has been reported [28–30], and technical factors are fully considered as causes of anastomotic leakage and local recurrence. We practice total mesorectal excision [31, 32] with the utmost care not to destroy the fascia propria of rectum, and we believe that this is one of the factors that did not create an association between anastomotic leakage and recurrence rate and survival rate.

This study has several limitations. First, this was a single-institution retrospective analysis. Second, biases were introduced due to the lack of randomization or propensity scoring. Third, all the dissection group cases were open, whereas many of the non-dissection group cases were laparoscopic; moreover, the dissection group was considerably older than the non-dissection group. In the future, more accurate methods for extracting cases that would benefit from prophylactic LLND and the administration of preoperative radiotherapy are warranted. Furthermore, we need to be cautious about performing unnecessary prophylactic LLND procedures that could deteriorate patients’ postoperative quality of life.

## Conclusion

In conclusion, this study demonstrated that the effectiveness of prophylactic LLND was limited in patients with > T3 rectal cancers with no evidence of preoperative lymph node metastasis. Prophylactic LLND may not be necessary if there is no preoperative lymph node metastasis, even if the invasion depth is T3 or higher.

## Data Availability

The datasets generated and/or analyzed during the current study are not publicly available due to patient privacy and security of electronic medical information but are available (anonymized) from the corresponding author on reasonable request.

## Abbreviations

ASA-PS: American Society of Anesthesiologists physical status
CT: Computed tomography
LLND: Lateral lymph node dissection
ME: Mesorectal excision
OS: Overall survival
RFS: Relapse-free survival
